# The relationship between the preoperative systemic inflammatory response and cancer-specific survival in patients undergoing potentially curative resection for renal clear cell cancer

**DOI:** 10.1038/sj.bjc.6603034

**Published:** 2006-03-07

**Authors:** G W A Lamb, D C McMillan, S Ramsey, M Aitchison

**Affiliations:** 1 Department of Urology, Gartnavel General Hospital, Glasgow G12 0YN, UK; 2 University Department of Surgery, Royal Infirmary, Glasgow G31 2ER, UK

**Keywords:** renal cancer, nephrectomy, tumour stage, grade, performance status, systemic inflammatory response, cancer-specific survival

## Abstract

The relationship between tumour stage, grade (Fuhrman), performance status (ECOG), a combined score (UCLA Integrated Staging System, UISS), systemic inflammatory response (elevated C-reactive protein concentration), and cancer-specific survival was examined in patients undergoing potentially curative resection for renal clear cell cancer (*n*=100). On univariate survival analysis, sex (*P*=0.050), tumour stage (*P*=0.001), Fuhrman grade (*P*<0.001), UISS (*P*<0.001), C-reactive protein (*P*=0.002) were significant predictors of survival. On multivariate analysis with sex, UISS and C-reactive protein entered as covariates, only UISS (HR 2.70, 95% CI 1.00–7.30, *P*=0.050) and C-reactive protein (HR 4.00, 95% CI 1.21–13.31, *P*=0.024) were significant independent predictors of survival. The presence of a preoperative systemic inflammatory response predicts poor cancer-specific survival in patients who have undergone potentially curative resection for renal clear cell cancer.

Renal cell cancer, although the twelfth most common cause of cancer death is one of the most lethal urological cancers. Each year in the UK, there are approximately 3500 new cases and approximately 30% of patients present with metastases. Overall survival is poor; even in those who undergo potentially curative resection, only approximately half survive 5 years (Cancerstats, www.cancerresearchuk.org).

The ideal prognostic score for patients undergoing potentially curative resection of a primary renal cancer should clearly distinguish those who will eventually succumb to the disease from those who are cured. While TNM stage has been widely used, it fails to provide clear separation between these groups. This has lead to the development of a number of cumulative prognostic scores including TNM stage. TNM stage has been combined with tumour grade and performance status to form the UCLA Integrated Staging System (UISS, Zisman *et al*, 2002).

It is recognised that in addition to tumour stage and proliferative activity, disease progression is dependent on a complex interaction of the tumour and host inflammatory response (Balkwill and Mantovani, 2001; Coussens and Werb, 2002; Vakkila and Lotze, 2004). Recently, the systemic inflammatory response, as evidenced by elevated circulating concentrations of C-reactive protein, has been shown to be a stage independent prognostic factor in patients undergoing potentially curative resection for colorectal cancer (McMillan *et al*, 2003), pancreatic cancer (Jamieson *et al*, 2005) and urinary bladder cancer (Hilmy *et al*, 2005).

The aim of the present study was to examine the prognostic value of the systemic inflammatory response in patients undergoing potentially curative resection for renal cancer.

## PATIENTS AND METHODS

Patients with renal clear cell cancer, who, on the basis of surgical findings and preoperative computed tomography of chest abdomen and pelvis underwent potentially curative resection between August 1996 and November 2004 in the West of Scotland, were included in the study. No patient had metastatic disease and the tumour confined to the kidney. In the case of T3/T4 tumours all macroscopic tumour was removed with clear resection margins.

Patients were staged pathologically according to the 1997 UICC TNM classification of renal tumours (Sobin and Wittekind, 1997). Tumours were graded according to criteria set out by Fuhrman *et al* (1982). Tumour stage, grade and ECOG-ps were collated into the UISS (Zisman *et al*, 2002, Table 1).

Data for 1996–2000 (*n*=57) were collected retrospectively and that for 2001–2004 (*n*=43) prospectively. Clinical stage and performance status (Eastern Cooperative Oncology Group, ECOG-ps) were recorded prior to surgery. Also, routine laboratory measurement of C-reactive protein was carried out.

The Research Ethics Committee of North Glasgow NHS Trust approved the study.

Routine laboratory measurement of patient's serum for C-reactive protein concentration was performed. The limit of detection of the assay was a C-reactive protein concentration lower than 6 mg l^−1^. The coefficient of variation, over the range of measurement, was less than 5% as established by routine quality control procedures. C-reactive protein measurement of greater than 10 mg l^−1^ was considered to indicate the presence of systemic inflammatory response (O'Gorman *et al*, 2000).

### Statistics

Comparisons between groups of patients were carried out using contingency table analysis (*X*^2^) as appropriate. Survival analysis was performed using the Cox's proportional-hazards model. Deaths up to the end of October 2005 were included in the analysis. Multivariate survival analysis was performed using a stepwise backward procedure to derive a final model of the variables that had a significant independent relationship with survival. To remove a variable from the model, the corresponding *P*-value had to be greater than 0.10. Analysis was performed using SPSS software (SPSS Inc., Chicago, IL, USA).

## RESULTS

The characteristics of patients with renal cancer who underwent potentially curative resection (*n*=100) are shown in Table 2. The majority was male, over the age of 60 years, had good performance status, low T stage (median tumour volume 178 cm^3^) and were defined as UISS intermediate risk. Approximately, 40% had an elevated C-reactive protein concentration (>10 mg l^−1^).

The minimum follow-up was 12 months; the median follow-up of the survivors was 59 months. During this period 25 patients died; 18 patients of their cancer and seven of intercurrent disease. On univariate survival analysis, sex (*P*=0.050), tumour stage (*P⩽*0.001), Fuhrman grade (*P*<0.001), UISS (*P*<0.001) and C-reactive protein (*P*<0.01) were significant predictors of cancer-specific survival.

On multivariate analysis with sex, tumour stage, Fuhrman grade, performance status and C-reactive protein entered as covariates, only sex (HR 0.25, 95% CI 0.06–0.99, *P*=0.048), Fuhrman grade (HR 2.91, 95% CI 1.29–6.56, *P*=0.010) and C-reactive protein (HR 7.67, 95% CI 1.64–35.84, *P*=0.010) were significant independent predictors of cancer-specific survival.

On multivariate analysis with sex, UISS and C-reactive protein entered as covariates, only UISS (HR 2.70, 95% CI 1.00–7.30, *P*=0.050) and C-reactive protein (HR 4.00, 95% CI 1.21–13.31, *P*=0.024) were significant independent predictors of cancer-specific survival. A greater proportion of females compared with males had Fuhrman grade I tumours (10 *vs* 29%, *P*=0.030, Fisher's exact test).

The relationship between the systemic inflammatory response and the clinicopathological characteristics are shown in Table 3. There was no significant difference in age or sex between the inflammatory and noninflammatory groups. An elevated C-reactive protein was associated with a greater number of patients with advanced tumour stage (*P*<0.01), increased grade (*P*<0.05), poorer performance status (*P*<0.01) and consequently a high UISS (*P*<0.01). Those patients with an elevated preoperative C-reactive protein concentration (>10 mg l^−1^) had a mean cancer-specific survival of 71 months compared with 96 months (*P*<0.001) in those patients with a C-reactive protein concentration in the normal range (⩽10 mg l^−1^).

**Figure 1:**
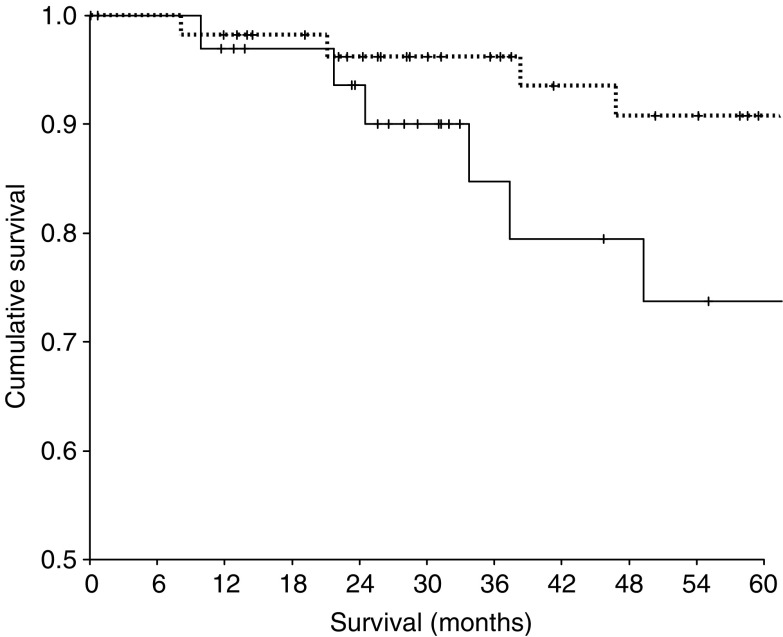
Relationship between preoperative C-reactive protein (⩽10/>10 mg l^−1^ from top to bottom) and cancer-specific survival in ‘low’ or ‘intermediate risk’ patients (*n*=91) undergoing potentially curative resection for renal cancer.

In those patients with a UISS risk classified as ‘low’ or ‘intermediate’ (*n*=91) an elevated C-reactive protein concentration was associated with a decrease in cancer-specific survival (*P*=0.008, Figure 1).

## DISCUSSION

Surgical resection remains the only prospect for long-term survival in patients with renal clear cell cancer. Currently, in patients undergoing surgery prognosis is based on tumour stage and grade and performances status (UISS, Zisman *et al*, 2002). In the present study, the preoperative measurement of C-reactive protein provided additional prognostic information.

To our knowledge this is only the second study to examine the role C-reactive in predicting survival following potentially curative resection for renal cancer. Masuda *et al* (1998) in a retrospective study of 111 patients reported that C-reactive protein was a prognostic factor independent of tumour stage and grade. However, performance status was not considered and thresholds for C-reactive protein were not defined in the survival analysis.

Few studies have identified factors which give prognostic information in addition to the UISS criteria, namely T stage, Fuhrman grade and ECOG-ps, in patients undergoing potentially curative resection for renal cancer. Recently, Shvarts *et al* (2005) reported that tumour p53 expression was related to Fuhrman grade and displaced it in multivariate analysis. Similarly, Lam *et al* (2005) reported that tumour Ki-67, unlike carbonic anhydrase, was related to the degree of necrosis and had independent prognostic value. Further studies are required to determine whether an elevated C-reactive protein has prognostic value independent of these other biological factors, in particular Ki-67.

It has been previously shown that, in patients undergoing potentially curative surgery for colorectal and pancreatic cancer, approximately one-third and one-half respectively, of patients had elevated circulating concentration of C-reactive protein preoperatively and that these patients had a significantly lower cancer-specific survival (McMillan *et al*, 2003; Jamieson *et al*, 2005). It was of interest that, in the present study, the proportion of patients with an elevated preoperative C-reactive protein concentration was similar and that these patients also had a poorer outcome.

It may be that because C-reactive protein concentration has prognostic value independent of UISS criteria, it might be added to these criteria to improve the prediction outcome, in particular the large group of ‘low or intermediate risk’ patients with renal cancer. Indeed, this approach has recently been used to improve the prediction of outcome in patients who underwent potentially curative resection for colorectal cancer (Canna *et al*, 2004).

An elevated circulating C-reactive protein concentration is also an established poor prognostic factor in patients with metastatic renal cancer (Atzpodien *et al*, 2003; Bromwich *et al*, 2004; Casamassima *et al*, 2005). In our previous study of patients with metastatic renal cancer (Bromwich *et al*, 2004), approximately 70% had an elevated C-reactive protein (>10 mg l^−1^) compared with approximately 40% in the present study of primary operable disease. This is consistent with previous observations that the systemic inflammatory response increases with advancing disease (Mahmoud and Rivera, 2002). However, the basis of the independent relationship between an elevated C-reactive protein concentration and poor survival in renal cancer is not clear. There are a number of possible explanations. Firstly, that an elevated C-reactive protein identifies tumours capable of producing significant amounts of proinflammatory cytokines, in particular interleukin-6 (Kinoshita *et al*, 1999; McKeown *et al*, 2004) and therefore with the potential for more rapid growth of tumour cells (Jee *et al*, 2001; Trikha *et al*, 2003). Also, that an elevated C-reactive protein identifies those patients with T-lymphocyte impairment (Maccio *et al*, 1998; Canna *et al*, 2005) or patients with a proangiogenic environment (Kofler *et al*, 2005; Xavier *et al*, 2006) allowing unrestrained tumour growth and dissemination. Clearly, both these tumour and host mechanisms may be related and required for the greater malignant potential associated with an elevated C-reactive protein concentration.

This is a relatively small, partially retrospective study and requires verification in larger prospective cohorts. However, if an elevated C-reactive protein concentration is shown to offer prognostic value in addition to the current UISS criteria it would improve our staging of these patients. Moreover, C-reactive protein may offer a useful preoperative therapeutic target in patients undergoing potentially curative surgery for renal clear cell cancer.

In summary, the presence of a preoperative systemic inflammatory response predicts poor cancer-specific survival in patients who have undergone potentially curative resection for renal clear cell cancer.

## Figures and Tables

**Table 1:**
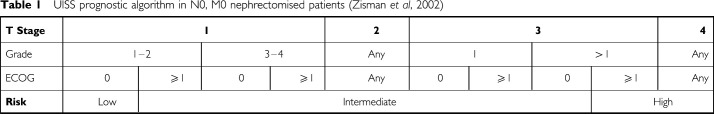
UISS prognostic algorithm in N0, M0 nephrectomised patients (Zisman *et al*, 2002)

**Table 2:** The relationship between clinicopathological characteristics and cancer-specific survival in patients undergoing potentially curative resection for renal cancer

	**Patients (*n*=100)**	**Hazard ratio (95% CI)**	***P*-value**
Age group (⩽60/>60 years)	43/57	1.48 (0.57–3.83)	0.419
Sex (male/female)	59/41	0.29 (0.08–1.00)	0.050
T stage (1/2/3/4)	36/19/42/3	4.33 (1.78–10.50)	0.001
Fuhrman grade (I/II/III/IV)	18/39/26/13	3.07 (1.67–5.67)	<0.001
ECOG-ps (0/⩾1)	86/14	2.56 (0.91–7.23)	0.075
UISS (low/intermediate/high)	22/69/9	5.72 (2.24–14.61)	<0.001
			
C-reactive protein (⩽10/>10 mg l^−1^)	58/42	5.85 (1.92–17.82)	0.002
			
Alive/dead	75/25		
Cancer-specific/intercurrent disease	18/7		

**Table 3:** The relationship between the presence of a preoperative systemic inflammatory response and clinicopathological characteristics of renal clear cell cancer

	**C-reactive protein**	**C-reactive protein**	
	**⩽10 mg l^−1^ (*n*=58)**	**>10 mg l^−1^ (*n*=42)**	***P*-value**
Age group (⩽60/>60 years)	26/32	17/25	0.664
Sex (male/female)	36/22	23/19	0.463
Tumour stage (1/2/3/4)	28/12/18/0	8/7/24/3	0.003
Fuhrman grade (I/II/III/IV)	10/28/14/3	8/11/12/10	0.021
ECOG-ps (0/⩾1)	54/4	32/10	0.016
UISS (low/intermediate/high)	17/40/1	5/29/8	0.003
			
Cancer-specific survival (months)^*^	95.9 (89.8–102.0)	70.8 (57.8–83.8)	<0.001

^*^Mean (95% CI).
